# Transforming growth factor‐β blocks glucose‐induced inflammation and apoptosis in corneal epithelial cells

**DOI:** 10.1002/2211-5463.12529

**Published:** 2018-11-06

**Authors:** Fengge Liu, Hui Kong, Xiangfeng Kong

**Affiliations:** ^1^ Department of Ophthalmology Zoucheng People's Hospital China

**Keywords:** apoptosis, cornea, glucose, inflammation, transforming growth factor‐β

## Abstract

Diabetic retinopathy is the most important ocular complication associated with diabetes. Corneal defects due to diabetes mellitus (DM) may cause severe vision impairments. This study aimed to identify the effect of transforming growth factor‐β (TGF‐β) on biological events, such as apoptosis and inflammation, in the diabetic cornea. High‐glucose treatment induced reactive oxygen species (ROS) production and several biological events, including apoptosis and inflammatory cytokine secretion, in human corneal epithelial cells. However, administration of TGF‐β significantly decreased ROS production, Annexin V‐positive cells, and levels of inflammatory cytokines. Sprague Dawley rats were injected with streptozotocin (STZ) as a model of DM. Inflammatory cytokine secretion, apoptosis, and inflammation were all increased by STZ treatment. However, apoptosis and inflammation were markedly reduced following TGF‐β treatment. In conclusion, TGF‐β can ameliorate the enhancement of apoptosis and inflammation in diabetic cornea in *in vivo* and *in vitro*.

**Abbreviations**
DCFH‐DA: 2′,7′‐dichlorofluorescein diacetate
DM: diabetes mellitus
HCECs: human corneal epithelial cells
IL‐1β: interleukin‐1β
ROS: reactive oxygen species
STZ: streptozotocin
TGF‐β: transforming growth factor‐β
TNF‐α: tumor necrosis factor‐α

Diabetes is a major endocrine disease worldwide 1. Diabetic retinopathy is the most important ocular complication associated with diabetes 2. Nonetheless, diabetic keratopathy is also a common complication 3, 4. Among the well‐recognized corneal changes associated with diabetic keratopathy, epithelial wound healing and nerve function have been most widely studied, mainly because they are associated with neurotrophic ulcer formation and surface irregularities 3, 5, 6, 7. However, the exact pathophysiology of diabetic keratopathy remains unknown, and it is difficult to study the various aspects of this disease by focusing solely on wound healing and nerve function.

Transforming growth factor‐β (TGF‐β), a major epithelial–mesenchymal transition inducer, binds TGF‐βRII to facilitate TGF‐βRII phosphorylation 8, 9. Phosphorylated TGF‐βRII can induce the phosphorylation of Smad2 and Smad3, and the subsequent complex of Smad2, Smad3, and Smad4 is delivered into the nucleus 10, 11. TGF‐β is involved in a variety of processes in cornea and is known to have three isoforms in mammals 12, 13. However, the effect of TGF‐β on the enhanced apoptosis and inflammation observed in the diabetic cornea has not been elucidated.

Hence, we hypothesized that the high‐glucose environment in diabetes enhances apoptosis and inflammation by promoting reactive oxygen species (ROS) production in the cornea and that this effect can be reversed by TGF‐β. Therefore, we aimed to identify the mechanism through which TGF‐β functions in the diabetic cornea using human corneal epithelial cells (HCECs) and streptozotocin (STZ)‐induced diabetic rat corneas.

## Materials and methods

### Cell culture

Human corneal epithelial cells (Thermo Fisher Scientific, Irvine, CA, USA) were cultured in bronchial epithelial growth medium (Lonza, Williamsport, PA, USA) at 37 °C and 5% CO_2_. The medium contained insulin (5 mg·mL^−1^), bovine pituitary extract (0.13 mg·mL^−1^), gentamycin (50 mg·mL^−1^), amphotericin (50 ng·mL^−1^), hydrocortisone (0.5 mg·mL^−1^), triiodothyronine (6.5 ng·mL^−1^), transferring (10 ng·mL^−1^), human epidermal growth factor (5 ng·mL^−1^), retinoic acid (10 ng·mL^−1^), and bovine serum albumin (0.15 mg·mL^−1^) (all from Sigma, St. Louis, MO, USA).

### TGF‐β mRNA levels in HCECs

Human CECs were cultured in glucose‐added medium for 24 h; total RNA was then isolated using the RNeasy mini kit in accordance with the manufacturer's protocol (Qiagen, Germantown, MD, USA). First‐strand cDNA was synthesized from 0.5 μg total RNA using PrimeScript 1st strand cDNA Synthesis Kit (Clontech, Mountain View, CA, USA). qPCR was performed in triplicate using SYBR Premix Ex Taq (Takara, Dalian, Liaoning, China) and SsoFast™ Probes Supermix (Bio‐Rad Laboratories, Inc., Hercules, CA, USA), and a standard thermocycling procedure (35 cycles) was performed on a Bio‐Rad CFX96™ Real‐time PCR System (Bio‐Rad Laboratories, Inc.). PCR cycles were as follows: 95 °C for 2 min, followed by 35 cycles of 95 °C for 1 min, 60 °C for 1 min, and 72 °C for 1 min, and then extension at 72 °C for 10 min. The 2^−ΔΔCq^ method was used to analyze the relative changes in gene expression.

The concentrations of TGF‐β in the supernatant were determined using a human ELISA kit (Sigma) in accordance with the manufacturer's protocol.

### ROS assay

Human corneal epithelial cells were cultured for attachment and growth in a six‐well plate and then treated with glucose at varying concentrations for 24 h. Intracellular ROS levels were detected using 2′,7′‐dichlorofluorescein diacetate (DCFH‐DA; Life Technologies, Grand Island, NE, USA) during a 30‐min incubation in the dark.

### Apoptosis

In the presence or absence of human TGF‐β, HCECs were cultured in media containing glucose in varying concentrations for 24 h. Harvested cells were washed with PBS and then centrifuged to collect cells from the precipitate. Cells were incubated with Annexin V/PI for 15 min. Flow cytometric analysis was then carried out.

### Western blotting

Briefly, protein concentration was measured to facilitate the use of a standard protein concentration of 30 μg per sample, and proteins were boiled for denaturation. After SDS/PAGE, proteins on the gel were transferred electrically onto a PVDF membrane. Proteins on PVDF membranes were probed with primary antibodies overnight at 4 °C, washed with TBST (3 × 10 min), and then incubated with secondary antibody. The protein bands were visualized with ECL reagent (Santa Cruz Biotechnology, Santa Cruz, CA, USA).

### Multiplex cytokine analysis

Levels of the pro‐inflammatory cytokines in the supernatants of HCECs treated with high‐glucose or normal medium, in the presence or absence of TGF‐β, were determined with an inflammatory cytokine human magnetic 5‐plex panel (Thermo Fisher Scientific) using multiplex bead technology. The human inflammatory magnetic 5‐plex panel contains all required reagents and is intended for use with the Luminex 200 dual laser detection system and xPONENT software (Luminex, Austin, TX, USA).

### Animal model

Sprague Dawley 6‐week‐old male rats were obtained from Experimental Animal Center of Chinese Academy of Sciences in Shanghai and were divided into three groups: diabetic (*n* = 6), diabetic TGF‐β (*n* = 6), and age‐matched normal control (*n* = 6). STZ was used to induce diabetes mellitus (DM) in these rats. The control group received a citrate buffer. Twenty‐four hours later, the glucose level in the rats was measured. A glucose concentration > 300 mg·μL^−1^ in heparinized tail vein blood was considered a successful induction. All animals received their respective treatment via eye drops three times a day during the experimental period, beginning 1 day after the intraperitoneal injection of STZ or buffer and ending 21 days after the injection. The wild‐type and diabetic control groups received one drop (40 μL) of PBS three times a day. The diabetic TGF‐β group received one drop of rat TGF‐β (200 μg·mL^−1^) three times a day. The study was approved by the Committee on the Ethics of Animal Experiments of Zoucheng People's Hospital. All surgery was performed under sodium pentobarbital anesthesia, and all efforts were made to minimize suffering. The animal experiments were carried out strictly following the guidelines given by the committee.

### Statistical analysis

All data were presented as the mean ± standard deviation (SD). Student's *t*‐test was used to identify the significance of difference between two groups, and one‐way analysis of variance (ANOVA) was used for multiple groups.*P <* *0.05 was considered to be statistically significant. graphpad prism iv software was carried out for statistical analyses.

## Results

### ROS in HCECs exposed to high glucose levels

**Figure 1:**
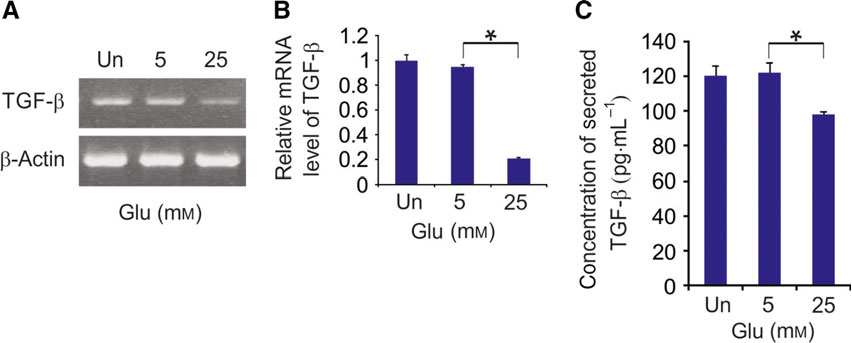
Reduced TGF‐β transcription and secretion in corneal epithelial cells cultured with high glucose concentrations (A) Expression of TGF‐β mRNA determined using semiquantitative RT‐PCR after glucose treatment. (B) Expression of TGF‐β mRNA determined using real‐time RT‐PCR after glucose treatment. The results are expressed as the means ± SD of three independent experiments. *P <* *0.05 (Student*'s t*‐test). (C) Production of TGF‐β protein, determined by ELISA, in HCECs cultured for 24 h in normal medium (control) or in medium containing normal (5 mm) or high glucose (25 mm). The results are expressed as the means ± SD of three independent experiments. *P <* *0.05 (Student*'s t*‐test).

First, the levels of TGF‐β mRNA and secreted TGF‐β were determined in HCECs exposed to normal‐glucose (5 mm) or high‐glucose (25 mm) medium for 24 h. Levels of both TGF‐β mRNA and secreted TGF‐β were significantly lower after exposure to high glucose levels (Fig. 1A–C).

**Figure 2:**
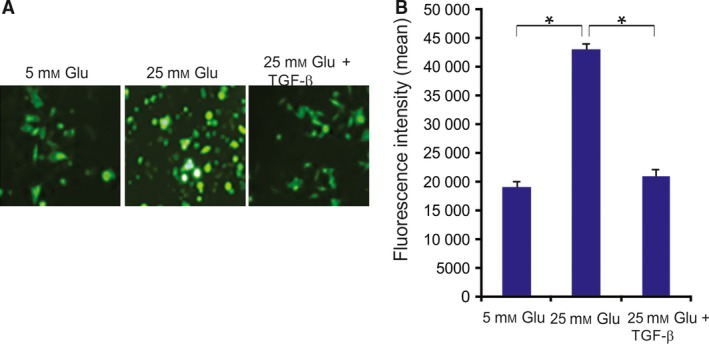
Fluorescence of DCFH‐DA oxidized by intracellular ROS (A) HCECs were cultured for 24 h in medium containing normal glucose (5 mm), high glucose (25 mm), or high glucose with TGF‐β (30 ng·mL^−1^). HCECs were then incubated with cell‐permeable DCFH‐DA (10 μm) for 45 min. Distribution of fluorescent DCF on the cell monolayer was photographed with a fluorescence microscope. (B) Quantitative flow cytometric measurements. The results are expressed as the means ± SD of three independent experiments. *P <* *0.05 (one‐way ANOVA with Tukey's *post hoc* test).

Next, we measured the ROS levels in HCECs exposed to high‐glucose medium. A higher number of HCECs exposed to high glucose levels (25 mm) exhibited high‐intensity fluorescence than did cells cultured at normal glucose levels (Fig. 2A,B). Furthermore, fluorescence intensity was greater in cells exposed to high glucose levels than in those incubated in normal glucose, a conclusion that was confirmed using fluorescence microscopy (Fig. 2A,B). The increased fluorescence intensity by high glucose levels was attenuated by TGF‐β treatment (Fig. 2A,B).

### Apoptosis in HCECs

**Figure 3:**
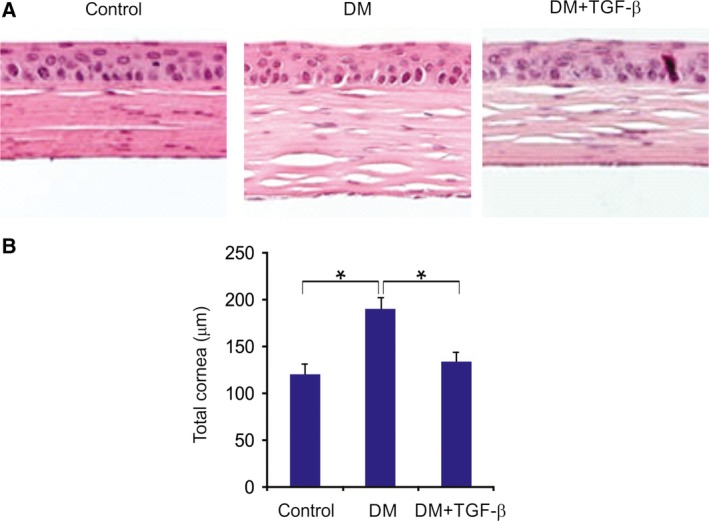
Corneal H&E staining and quantitative analysis of total corneal thickness (A) Histopathologic staining of diabetic rat cornea with the indicated treatment. (B) Mean differences in total corneal thickness in the control, diabetic, and diabetic with TGF‐β treatment groups. The results are expressed as the means ± SD of three independent experiments. *P <* *0.05 (one‐way ANOVA with Tukey's *post hoc* test).

In the corneal epithelium of the STZ‐induced diabetic rat, perinuclear clear areas and thickened basement membranes were observed. Furthermore, the corneal stroma was thicker due to edema. The total thickness of the diabetic cornea was significantly higher than that of the normal cornea. These changes in the diabetic cornea were consistently reversed by TGF‐β treatment (Fig. 3A,B).

**Figure 4:**
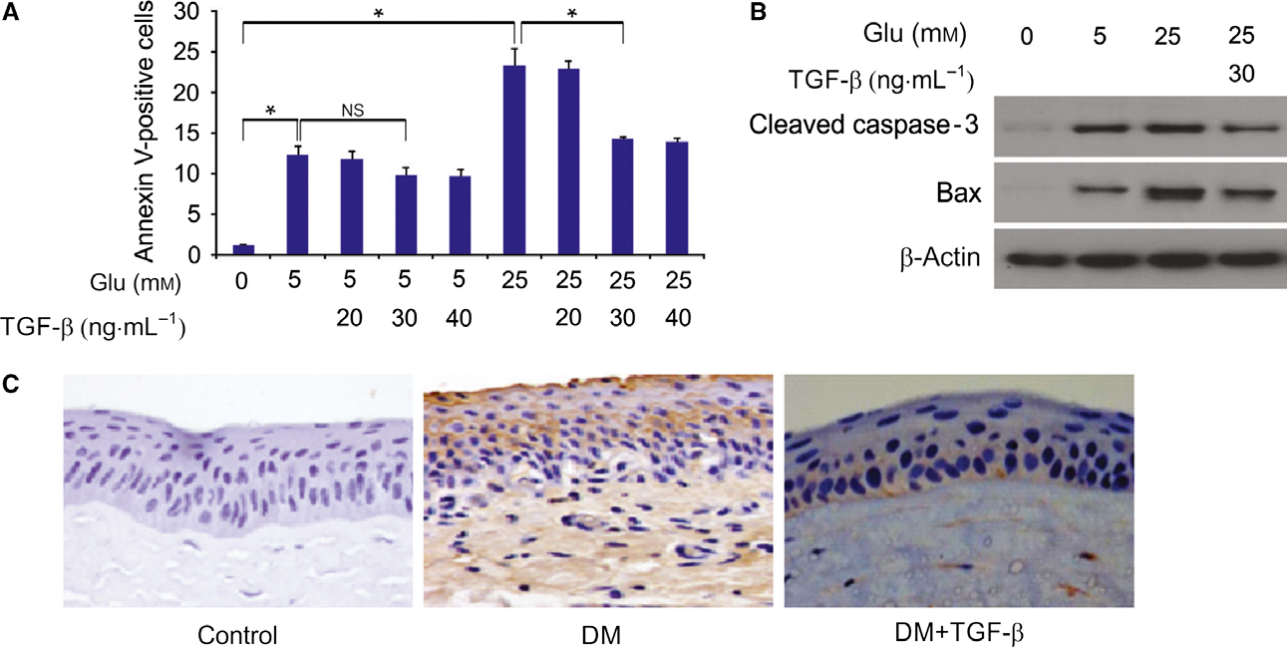
Cleaved caspase‐3 and BAX in glucose‐treated HCECs (A) Cells were treated for 24 h with normal glucose (5 mm), high glucose (25 mm), or high glucose with indicated concentration of TGF‐β. Cellular apoptosis was determined using flow cytometry. (B) Expression of cleaved caspase‐3 and Bax determined by western blotting after treatment for 24 h with the indicated treatment. (C) Cleaved caspase‐3 expression was determined by immunohistochemistry staining. The results are expressed as the means ± SD of three independent experiments. *P <* *0.05 (one‐way ANOVA with Tukey's *post hoc* test).

Next, we investigated cornea apoptosis in HCECs and STZ‐induced diabetic rat. The percentage of FITC‐positive cells varied depending on media, and a substantial difference was observed between cells cultured at normal glucose levels (12.3%) and high glucose levels (23.4%) (Fig. 4A). However, for cells receiving 30 ng·mL^−1^ TGF‐β treatment, the percentage of FITC‐positive cells decreased from 23.4% to 14.3%. These data imply that apoptosis had been partially suppressed by TGF‐β treatment (Fig. 4A). Western blot analysis showed significant increases in the concentrations of cleaved caspase‐3 and Bax in a high‐glucose environment (Fig. 4B). However, TGF‐β markedly suppressed the cleaved caspase‐3 and Bax increases in cells with a high‐glucose treatment (Fig. 4B). As shown in Fig. 4C, cleaved caspase‐3 expression increased in diabetic rat, which was inhibited by TGF‐β treatment.

### Inflammation in HCECs and rat corneas

**Figure 5:**
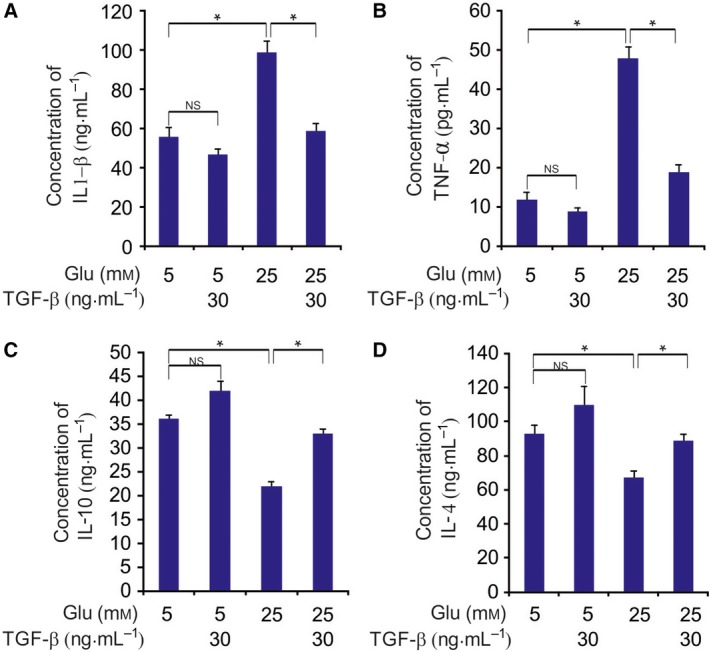
TGF‐β prevents high glucose‐induced inflammation in HCECs. (A) After identical treatment, IL‐1β expression was quantified using multiplex cytokine analysis. (B) After identical treatment, TNF‐α expression was quantified using multiplex cytokine analysis. (C) After identical treatment, IL‐10 expression was quantified using multiplex cytokine analysis. (D) After identical treatment, IL‐4 expression was quantified using multiplex cytokine analysis. The results are expressed as the means ± SD of three independent experiments. *P <* *0.05 (one‐way ANOVA with Tukey's *post hoc* test).

We next examined whether TGF‐β could reduce the levels of secreted interleukin‐1β (IL‐1β) and tumor necrosis factor‐α (TNF‐α). HCECs exposed to high glucose levels secreted significantly more TNF‐α and IL‐1β than those incubated in normal glucose levels (Fig. 5A,B). TGF‐β treatment significantly reduced the levels of secreted TNF‐α and IL‐1β in high‐glucose medium (Fig. 5A,B). In addition, HCECs exposed to high glucose levels secreted significantly less IL‐10 and IL‐4 than those incubated in normal glucose levels (Fig. 5C,D). TGF‐β treatment significantly increased the levels of secreted IL‐10 and IL‐4 in high‐glucose medium (Fig. 5C,D).

**Figure 6:**
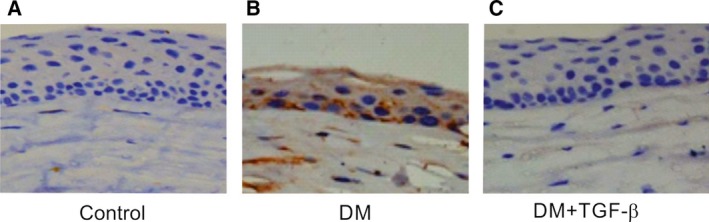
Immunohistochemistry staining of IL‐1β in the cornea. (A) Representative photomicrographs of the corneas of control rats. (B) Representative photomicrographs of the corneas of diabetic rats. (C) Representative photomicrographs of the corneas of diabetic rats after TGF‐β treatment .

Immunohistochemically, the epithelium, stroma, and endothelium of the diabetic cornea showed intense staining for IL‐1β (Fig. 6B; compare with the control in Fig. 6A). However, weak immunohistochemical staining for IL‐1β was observed in the diabetic cornea after TGF‐β treatment (Fig. 6C).

## Discussion

Previous studies have confirmed that dry eye symptoms in diabetes are correlated with the severity of the diabetic retinopathy 14, 15. In relation to this, several investigators have suggested that dry eye symptoms in diabetes are associated with peripheral polyneuropathy caused by reduced corneal sensitivity, altered homeostasis of the cornea, or ocular surface dysfunction caused by tear film instability and reduced tear secretion 16, 17, 18. Nevertheless, the exact pathophysiological changes that occur in the diabetic cornea remain controversial. Recently, several studies have suggested that the initial step in the immunopathogenic mechanism responsible for dry eye syndrome is desiccating stress on ocular surface epithelial cells 19, 20. The glucose concentration in tears correlates with blood glucose levels, as in dry eye syndrome 21. Therefore, we hypothesized that biochemical and ultrastructural changes in the cornea induced by high glucose levels, especially in the corneal epithelium, lead to desiccating stress and that such changes are the most important factor leading to dry eye in diabetic patients whose corneas appear disease‐free, more important than structural changes in the nerves or basement membrane.

It has been suggested that TGF‐β plays a role in ocular surface healing and sensitivity in corneal and conjunctival cells 22, 23, 24, 25. Therefore, we assessed TGF‐β mRNA and TGF‐β production in HCECs cultured in high glucose. Next, to assess whether high glucose levels enhance ROS, apoptosis, and inflammation in corneal epithelial cells, as well as whether TGF‐β can reverse these pathologic changes, we evaluated these phenotypes in HCECs cultured in high glucose with or without TGF‐β. To obtain *in vivo* evidence, we then investigated histopathologic, apoptotic, and inflammatory changes in the corneas of diabetic rats with or without TGF‐β treatment.

Our results demonstrate that TGF‐β mRNA and TGF‐β production are significantly reduced in HCECs cultured in high glucose, enhancing ROS production and increasing the quantity of apoptotic cells. High glucose levels significantly increase the levels of pro‐inflammatory cytokines in HCECs. That is, we have demonstrated that high glucose levels enhance ROS, apoptosis, and inflammation in HCECs. Furthermore, in the corneas of STZ‐induced diabetic rats, we discovered that mitochondria‐dependent caspase activity enhanced apoptosis. This study also demonstrated that enhanced inflammation is associated with increased IL‐1β. Unlike apoptosis, immunoreactivity in the form of IL‐1β was detected evenly across the entire cornea, suggesting that secreted inflammatory cytokines from damaged cells are distributed across the cornea. Nevertheless, the most important finding of our study is that TGF‐β can reverse all the diabetes‐associated phenomena both *in vitro* and *in vivo*.

Taken together, our results indicate that diabetic hyperglycemia enhances apoptosis and inflammation in the cornea, especially in the corneal epithelium, and that this occurs without any severe neurotrophic ulcerative changes. This may explain the unusually severe dry eye symptoms that occur in patients with diabetes whose corneas appear disease‐free. In addition, our results confirmed that TGF‐β can successfully suppress this enhanced apoptosis and inflammation in the diabetic cornea. However, the current study is the first to demonstrate that TGF‐β can ameliorate the apoptosis and inflammation in the diabetic cornea with both *in vitro* and *in vivo* experiments.

## Conclusions

In summary, high glucose in diabetes induces apoptosis and inflammation in the cornea by promoting ROS formation. These changes are most apparent in the corneal epithelial cells. Furthermore, the biochemical changes associated with apoptosis and inflammation occur without severe structural change, and this may explain the dry eye symptoms seen in diabetic patients. We have provided evidence that these pathologic changes can be reversed with TGF‐β treatment. Hence, TGF‐β could be used to treat mild‐to‐severe diabetic keratopathy, and targeted prevention of apoptosis may represent a novel therapeutic approach to managing the diabetic cornea.

## Author contributions

FL and HK conceived the study and designed the experiments. FL and XK contributed to the data collection. HK and XK performed the data analysis and interpreted the results. FL wrote the manuscript. HK contributed to the critical revision of article. All authors read and approved the final manuscript.

## Conflict of interest

The authors declare no conflict of interest.
